# Editorial: Environmental pollutants in agroecosystem: toxicity, mechanism, and remediation

**DOI:** 10.3389/fpls.2023.1208405

**Published:** 2023-06-07

**Authors:** Muhammad Musa Khan, Pankaj Bhatt

**Affiliations:** ^1^ Plant Inspection, Quarantine and Crop Health Team, Hainan Institute of Zhejiang University, Sanya, China; ^2^ Department of Agricultural & Biological Engineering, Purdue University, West Lafayette, IN, United States

**Keywords:** environmental pollution, plants, vertebrates, invertebrates, biological and physiological stress, heavy metals, pesticides, microplastics

## 1 Introduction

Environmental contamination in agroecosystems is a significant problem caused by various pollutants that can impact soil, water, air, and the surrounding ecosystem. Agroecosystems are complex systems where agriculture, ecology, and the environment interact, and contamination in one aspect can have cascading effects on the entire system (Wang et al., 2020). Contaminants in agroecosystems can come from various sources, including agricultural practices, industrial activities, and urbanization (Singh and Sharma, 2021).

Some agroecosystems’ most common environmental contaminants are heavy metals, pesticides, and emerging pollutants (Picó et al., 2020). Heavy metals such as cadmium, lead, and mercury among others can accumulate in the soil and crops, leading to potential health hazards (Zwolak et al., 2019). Pesticides, including herbicides, insecticides, and fungicides, can also contaminate soil, water, and air, adversely affecting non-target organisms (Li et al., 2023). Emerging pollutants, such as pharmaceuticals and personal care products, are a growing concern in agroecosystems, as they can persist in the environment and pose long-term risks to ecosystem health (Osuoha et al., 2023). As different environmental pollutants have attracted attention, potential remediation techniques and methods have developed rapidly (Sun et al., 2018).

In this editorial, we set up a Research Topic of *Environmental pollutants in an agroecosystem: Toxicity, Mechanism, and Remediation*, which covers not only environmental pollutants in agroecosystems but also in the aquatic environment. The following themes are included in this Research Topic: (a) Risk assessment of environmental pollutants to plants; (b) Biological alterations induced by pollutants in plants and invertebrates; (c) Physiological and molecular mechanism of plants and vertebrates against pollutants; (d) Environmental pollutants as a risk to agricultural practices; (e) Remediation techniques for environmental pollutants in the field.

Despite significant advances in understanding the environmental consequences of environmental pollutants, there remain knowledge gaps in these areas, and our Research Topic aims to address these gaps. In the end, we accepted and published 10 articles written by 99 researchers from seven different countries, such as China, India, Saudi Arabia, Pakistan, South Korea, Portugal, and the United Kingdom.

## 2 Remediation of the toxic pollutants

Remediation of toxic pollutants is an important process to protect human health and the environment by eliminating or mitigating the harmful effects of these substances. Various methods are available for remediating toxic pollutants, depending on the type and severity of contamination. Here are some common techniques which were reported in this research Topic:

- Bioremediation: this technique uses living organisms, such as bacteria, fungi, and plants, to break down, remove, or neutralize contaminants in soil, water, and air. The organisms may be naturally occurring or genetically engineered to enhance their remediation capabilities.
- Chemical treatment: chemicals react with pollutants, neutralizing or transforming them into less harmful substances. Examples include oxidation, reduction, precipitation, and neutralization processes.
- Phytoremediation: this technique uses plants to absorb, accumulate, or break down pollutants in the soil, water, or air. Some plants, such as sunflowers, are particularly effective at extracting heavy metals and other contaminants.

-Zhao et al. reported that foliar silicon (Si) spraying could reduce rice’s cadmium (Cd) contamination, but different rice varieties respond differently. Si-inhibited varieties show a decrease in Cd content by 13.5%–65.7%, while Si-stimulated varieties experience an increase of 15.7%–24.1%. This highlights the importance of considering rice variety differences when implementing foliar Si spraying to remediate Cd-contaminated paddy fields.

-Lei et al. reported silicon's role in mitigating cadmium toxicity in plants. Silicon helps reduce cadmium uptake and transport, improves nutrient supply, regulates antioxidant systems, and enhances physical plant structure. The review specifically focuses on silicon's role in maintaining water balance and suggests future research directions.

-Huang et al. duckweed have phytoremediation ability and stated that high streptomycin concentrations negatively impact duckweed health, reducing biomass and growth rate while increasing antioxidant enzymes. However, duckweed demonstrates a high ability to remove streptomycin from the environment, with significant reductions observed after 20 days. This suggests that duckweed could be a valuable resource for treating aquaculture wastewater and domestic sewage contaminated with streptomycin.

-Zhu et al. stated that antioxidant enzymes and non-enzymatic antioxidants increased with Pb concentration, while peroxidase and the ascorbic acid-glutathione cycle showed mixed results. Through transcriptome sequencing, 17 root Pb-tolerant genes were identified, associated with antioxidant, transport, and transcription functions.

-Hafeez et al. reported that different plant hosts affected the bio-detoxification of insects differently by affecting the gene expression of a specific gene. This not only affects the efficiency of an insecticide against a pest but could also contribute to the development of insecticide resistance. The results suggested that the P450 enzyme system helps the herbivores adapt to the diverse host plant by developing different secondary compounds in their hosts.

-The study presented by Ejaz et al. focuses on the mechanism of heavy metals uptake in plant and their detoxification. The results stated that heavy metal concentrations exceeding permissible limits seriously threaten humans, plants, and other life forms. Plants absorb these toxic metals and employ strategies to cope with the contamination, such as restricting heavy metals within cell walls or synthesizing compounds to bind metal ions. They highlighted the importance of studying model plant species’ genetics, molecular, and cell signaling aspects to understand their heavy metal tolerance strategies and potentially apply that knowledge to mitigate the negative effects of heavy metal contamination.

## 3 Rhizo-microbiome in environmental sustainability

The rhizo-microbiome, also known as the rhizosphere microbiome, is a complex community of microorganisms that resides in the soil surrounding plants roots i.e. rhizosphere. This diverse group of microbes, including bacteria, fungi, and archaea, is vital in promoting plant health, growth, and overall ecosystem functioning. Understanding and harnessing the potential of the rhizo-microbiome can greatly contribute to environmental sustainability. Some of how the rhizo-microbiome supports sustainability include:

- Nutrient cycling: rhizo-microbes play a crucial role in nutrient cycling, including nitrogen fixation, phosphorus solubilization, and potassium release. By converting these nutrients into plant-available forms, the rhizo-microbiome helps reduce the need for chemical fertilizers, which can have negative environmental impacts.
- Soil health and structure: rhizo-microbes contribute to soil aggregation and improve soil structure, which can prevent soil erosion, promote water infiltration, and enhance overall soil health. Healthy soils are essential for sustainable agriculture and long-term ecosystem stability.
- Phytoremediation: some plants, in association with their rhizo-microbes, can uptake and degrade pollutants from the soil, known as phytoremediation. This can help restore contaminated lands and improve overall environmental quality.

Rhizo-microbiome plays a vital role in maintaining and enhancing environmental sustainability. By understanding and harnessing the potential of these microbial communities, we can develop more sustainable agricultural practices, protect and restore ecosystems, and mitigate the impacts of climate change.

-The research conducted by Patani et al. reported that out of 107 PGPR strains, five *Bacillus* strains significantly improve tomato plant growth and productivity. Inoculated salt-stressed tomato plants had higher levels of essential nutrients and antioxidant enzyme activity while maintaining lower levels of harmful ions, indicating that halotolerant PGPR strains can mitigate the negative effects of salt stress on tomato plants.

-Li et al. explained how *Dahlia pinnata* accumulate Cd and detoxify heavy metal and the role of rhizospheric microbiota in phytoremediation.

## 4 Future research

In summary, the articles published in this Research Topics suggest that the research conducted thus far in these topics has significantly improved our comprehension of the environmental fate, ecotoxicology, risk assessment, and remediation of various pollutants. However, considerable challenges persist in the realm of computational toxicology, particularly in predicting the environmental risks posed by pollutants and understanding the combined effects of various contaminants (such as heavy metals, PPCPs, and microplastics). Further investigation is necessary in these domains, with a particular emphasis on the transfer of pollutants across different trophic levels, their biodegradation, and the mechanisms behind their impact.

## Author contributions

MK authored the initial draft of this editorial, which PB subsequently revised and approved for submission. As guest topic editors, both MK and PB have been heavily involved in the call for submissions and have overseen the editing process for the manuscripts submitted to this Research Topic.
